# Residual Primary Breast Cancer After Chemotherapy: Assessment of Recurrence and Survival

**DOI:** 10.7759/cureus.107973

**Published:** 2026-04-29

**Authors:** Nicolas Ramírez-Torres, Rodolfo Rivas-Ruiz

**Affiliations:** 1 Gynecology and Obstetrics, Centro Médico Nacional La Raza, Instituto Mexicano del Seguro Social, Mexico City, MEX; 2 Clinical Research Division, Coordinación de Investigacion en Salud, Instituto Mexicano del Seguro Social, Mexico City, MEX

**Keywords:** breast neoplasms, neoadjuvant therapy, prognosis., recurrence, survivall

## Abstract

Introduction

The presence of residual breast cancer after neoadjuvant chemotherapy can influence the rate of distant recurrence.

Objective

To identify clinical and tumoral risk factors predicting recurrence, and to evaluate their impact on distant recurrence-free survival (DRFS), in patients with stage III breast cancer who did not achieve a pathological complete response (non-pCR) after neoadjuvant chemotherapy

Material and methods

This retrospective study evaluated the association between risk factors and recurrence in patients who did not achieve a pathological complete response using relative risk. Five-year distant recurrence-free survival was calculated for three risk groups: low-risk, intermediate-risk, and high-risk. The discriminative ability for predicting recurrence was assessed using receiver operating characteristic methodology. Two Cox regression analyses were performed to identify independent predictors of relapse.

Results

Residual tumor was present in 69.8% (88/126) of patients, with 47 total recurrences. Significant risk factors included clinical stage IIIB, grade 3 tumors, and luminal B and HER2+ subtypes, with relative risks (RRs) of 3.5 (95% CI: 1.6-7.5), 3.7 (95% CI: 1.7-8), 3.4 (95% CI: 1.3-8.6), and 4.2 (95% CI: 1.1-15), respectively. Recurrence rates for the low-risk, intermediate-risk, and high-risk groups were 22% (10/45), 36% (19/53), and 64% (18/28), respectively, with corresponding five-year distant recurrence-free survival rates of 67% (95% CI: 43-83%), 61% (95% CI: 46-73%), and 36% (95% CI: 18-54%). The positive likelihood ratios were 0.38 for the low-risk group, 0.71 for the intermediate-risk group, and 2.94 for the high-risk group. Cox regression analysis showed that the intermediate-risk and high-risk groups had an increased risk for recurrence of 3.3 (95% CI: 1.2-9) and 5.4 (95% CI: 1.9-15) times, respectively, compared to the low-risk group.

Conclusions

This staging that includes a clinical and tumoral characteristics scale facilitates the early optimization of subsequent systemic treatments, allowing for therapeutic adjustments prior to the development of clinical metastasis.

## Introduction

Breast cancer is commonly diagnosed at advanced stages in Latin America and the Caribbean (30-60%), in contrast with 8.3-23.5% in developed countries in Europe and North America [1, 2]. In Mexico, the mortality rate from breast cancer has increased from 5.22 per 100,000 in 1970 to 9.65 per 100,000 in 2021 [3]. Late diagnosis of breast cancer, lack of adequate medical centers, treatment facilities, and social inequality contribute to a high mortality rate [3, 4]. Since 2006, women with breast cancer have accounted for more deaths than cervical cancer and other cancers [5].

Neoadjuvant chemotherapy (NAC) is essential in the management of high-risk localized, locally advanced, or unresectable breast cancer. The main goals are to maximally reduce the size of the primary tumor and eradicate affected lymph nodes for surgical purposes, prevent progression of micrometastases, and obtain early information about clinical and pathological response after NAC [6, 7].

In general, systemic drugs show initial activity in 90% of primary breast cancers and in 50% of metastatic cases [8]. Pathological complete response (pCR) is uncommon in patients with stage II and III breast cancer [9, 10], and micrometastases may already be present at diagnosis. Around 30% of patients with early-stage breast cancer experience distant recurrence (DR) [8].

Patients in stage II and III with residual tumor in the breast and axilla after chemotherapy and surgery are at high risk of developing regional or distant recurrence, or both, compared to those who achieved a pCR [8, 11]. These findings support the hypothesis: breast cancer is a systemic disease from its initial presentation.

There are different methodologies to measure and stratify tumor response following NAC [12-14]. Clinical and pathological factors can be combined to estimate the likelihood of breast cancer recurrence.

The primary objective of this study is to identify baseline clinical and tumoral risk factors that predict distant recurrence in patients with stage III breast cancer who do not achieve a pathological complete response (non-pCR) following neoadjuvant chemotherapy. The secondary objective is to evaluate the association between these combined risk factors and five-year distant recurrence-free survival (DRFS) by stratifying patients into low-, intermediate-, and high-risk groups.

## Materials and methods

Study population

This retrospective and longitudinal study included women under 75 years of age who were evaluated with the standard TNM Classification System, co-developed by the American Joint Committee on Cancer (AJCC) and the Union for International Cancer Control (UICC) [15]. Patients were included if they (1) had an initial diagnosis of unilateral breast cancer at clinical stage (CS) IIIA, IIIB, or IIIC, and (2) were treated between January 2009 and December 2015 with 5-fluorouracil, epirubicin, and cyclophosphamide (FEC), followed by sequential docetaxel (T) at the High Specialty Medical Unit of the Gynecology and Obstetrics Hospital No. 3, National Medical Center “La Raza,” affiliated with the Mexican Institute of Social Security.

Management

Oncological management with NAC and surgery, along with imaging and laboratory studies, was carried out at the study center and has been previously described [16]. The following variables were recorded: age, tumor size (T), axillary lymph nodes (LN), CS, tumor grade (G), histological type, molecular subtype, radiotherapy, and number of NAC cycles given. After histological diagnosis, the breast cancer was evaluated molecularly with distinct biomarkers using an immunohistochemical (IHC) assay for estrogen receptor (ER), progesterone receptor (PgR), and human epidermal growth factor receptor 2 (HER2) [9, 15].

Four molecular subtypes were identified: Luminal A (LA), Luminal B (LB), HER2-positive (HER2+), and triple negative (TN). Breast cancer staging was based on the American Joint Committee on Cancer (AJCC) tumor-node-metastasis (TNM) system [15].

Statistical analysis

The primary objective was to determine RFs for the prediction of recurrence before treatment in patients with stage III breast cancer. The secondary objective was to assess the association between risk groups and DRFS. Primary clinical outcomes of interest were recurrence after NAC and DRFS.

The clinical relevance and associations between RFs and recurrence were analyzed via chi-square tests to estimate relative risk (RR), 95% confidence intervals (CI), and p-values. Recurrence after NAC was recorded as: 0 = no DR, 1 = DR.

Based on the relative risk obtained in the bivariate model of significant independent RFs for recurrence of metastases, which were included in the clinical model, each factor was assigned its relative weight and summed in a Boolean model, accounting for a total of 11 categories for all the possible combinations (0 to 10). Each RF was analyzed with an ROC curve. Due to the small number of patients in some subgroups, the total score was divided into three groups for a better intergroup comparison: low risk (LR: 0), intermediate risk (IR: 1 to 6), and high risk (HR: 7 to 10) to improve the strength of the associations.

Significant RFs were included and combined in a clinical model to predict the risk of DR. Discriminative ability for recurrence was assessed using the receiver operating characteristic (ROC) curve. The point with the highest positive likelihood ratio (LR+) was estimated [17].

The hypothesis was that RFs must be combined to improve the predictive ability for recurrence in patients with residual tumors in the breast and axilla after chemotherapy. 

Survival was estimated after dividing risk groups for recurrence using the method of Kaplan-Meier, and the log-rank test was used to compare differences between groups. DRFS was calculated from the date of surgery to the date of recurrence or the fifth-year endpoint.

Two Cox regression analyses were performed to identify independent prognostic factors and risk groups associated with DRFS. The hazard ratio (HR), 95% CI, and p-values were estimated [18].

The significance of this study is that patients who did not achieve pCR were also evaluated; in contrast, other studies focus on pCR as the primary measure of response to systemic therapy in oncology practice [19, 20]. Written informed consent from patients was not required for this retrospective study. The study protocol was reviewed and accepted by the Local Research Committee with registry number R-2012-3504-24. All statistical tests were two-sided and performed using Stata version 14 (StataCorp., College Station, TX).

## Results

Characteristics of the study population

This study included 126 patients diagnosed with stage III breast cancer treated with NAC. Clinical-pathological and treatment data are shown in Table 1. The median diameter of the residual tumor after NAC was 2 cm (IQR: 0-3).

**Table 1:** Baseline characteristics of breast cancer patients and treatment characteristics (n=126) Continuous variables are presented as median (IQR), and categorical variables as n (%). Clinical stage was assigned according to the TNM (Tumor, Node, Metastasis) Classification System [15]. IQR: interquartile range (25th, 75th); ER: estrogen receptor; PgR: progesterone receptor; HER2: human epidermal growth factor receptor 2; NAC: neoadjuvant chemotherapy.

Characteristic	Value	%
Age, years	50.0 (44-58)	-
Initial tumor, cm	7.4 (6-9)	-
Final tumor, cm	2.0 (0-3)	-
Clinical tumor (T)		
T2	6	4.8
T3	61	48.4
T4	59	46.8
Clinical node (N)		
N1	56	44.4
N2	67	53.1
N3	3	2.5
Clinical stage		
IIIA	67	53.2
IIIB-C	59	46.8
ER expression level		
Absent	40	31.7
1-10%	6	4.7
11-50%	12	9.5
> 50%	68	53.9
PgR expression level		
Absent	61	48.4
1-10%	15	11.9
11-50%	24	19
> 50%	26	20.6
HER2		
Negative	101	80
Positive	25	20
Histology		
Ductal cancer	72	57.2
Lobular cancer	43	34.1
Other cancer	11	8.7
NAC, cycles		
< 6 cycles	7	5.6
> 6 cycles	119	94.4
Radiotherapy	110	87.3
Hormonal therapy	84	65.8

Bivariate analysis between risk factors and recurrence

**Figure 1:**
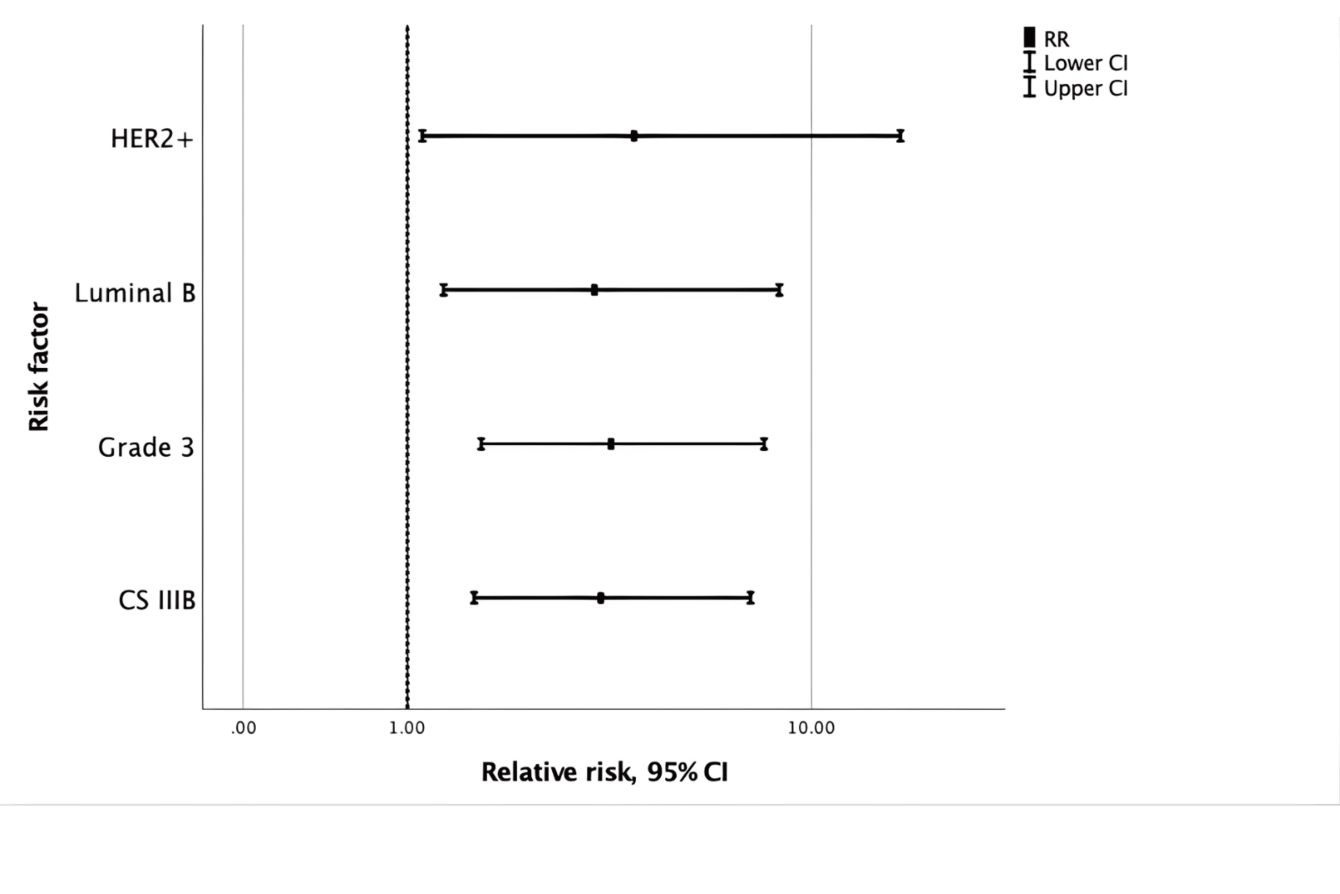
Bivariate analysis of risk factors for recurrence in breast cancer patients CS: Clinical Stage. Clinical stage was assigned according to the TNM (Tumor, Node, Metastasis) Classification System [15].

According to the bivariate analysis, RFs with statistically significant clinical relevance for recurrence were identified. CS IIIB, G3 tumors, and the LB and HER2+ subtypes had a RR of 3.5 (95% CI: 1.6 to 7.5), 3.7 (95% CI: 1.7 to 8), 3.4 (95% CI: 1.3 to 8.6), and 4.2 (95% CI: 1.1 to 15), respectively. Figure 1. Other parameters evaluated to predict risk of DR were age, ER, PgR, HER2, histology, NAC cycles, radiotherapy, and pCR, but none showed statistically significant clinical relevance.

The number and combination of independent RFs related to DR and the ROC curve coordinates are summarized in Table 2. At least 72.3% of patients had one RF. The DR prevalence in this cohort was 37.3% (37 with residual tumor and 10 with pCR).

**Table 2:** Individual risk factors for distant recurrence in breast cancer patients DR: distant recurrence; CS: clinical stage; G: tumor grade; LB: luminal B; HER2+: human epidermal growth factor receptor 2; Sen: sensitivity; Spe: specificity CS: Clinical Stage. Clinical stage was assigned according to the TNM (Tumor, Node, Metastasis) Classification System [15].

Value of the Scale	Independent risk groups	No DR		DR		Total	Curve coordinates		
		n = 79	%	n = 47	%	n = 126		Sen	Spe
0	No factor	35	77.7	10	22.2	45		0	0
1	CS IIIB	14	66.6	7	33.3	21		0.128	0
2	G3	3	75	1	25	4		0.149	0.038
3	CS IIIB/ G3	7	58	5	42	12		0.149	0.051
4	LB	3	75	1	25	4		0.383	0.127
5	LB/ CS IIIB	2	50	2	50	4		0.447	0.19
6	LB/ G3	5	62.5	3	37.5	8		0.489	0.215
7	LB/ G3/ CS IIIB/ HER2+	6	35.3	11	64.7	17		0.511	0.253
8	HER2+/ CS IIIB	1	100	0	0	1		0.617	0.342
9	HER2+/ G3	3	75	1	25	4		0.638	0.38
10	HER2+/G3/ CS IIIB	0	0	6	100	6		0.787	0.557

Discriminative ability for recurrence

**Figure 2:**
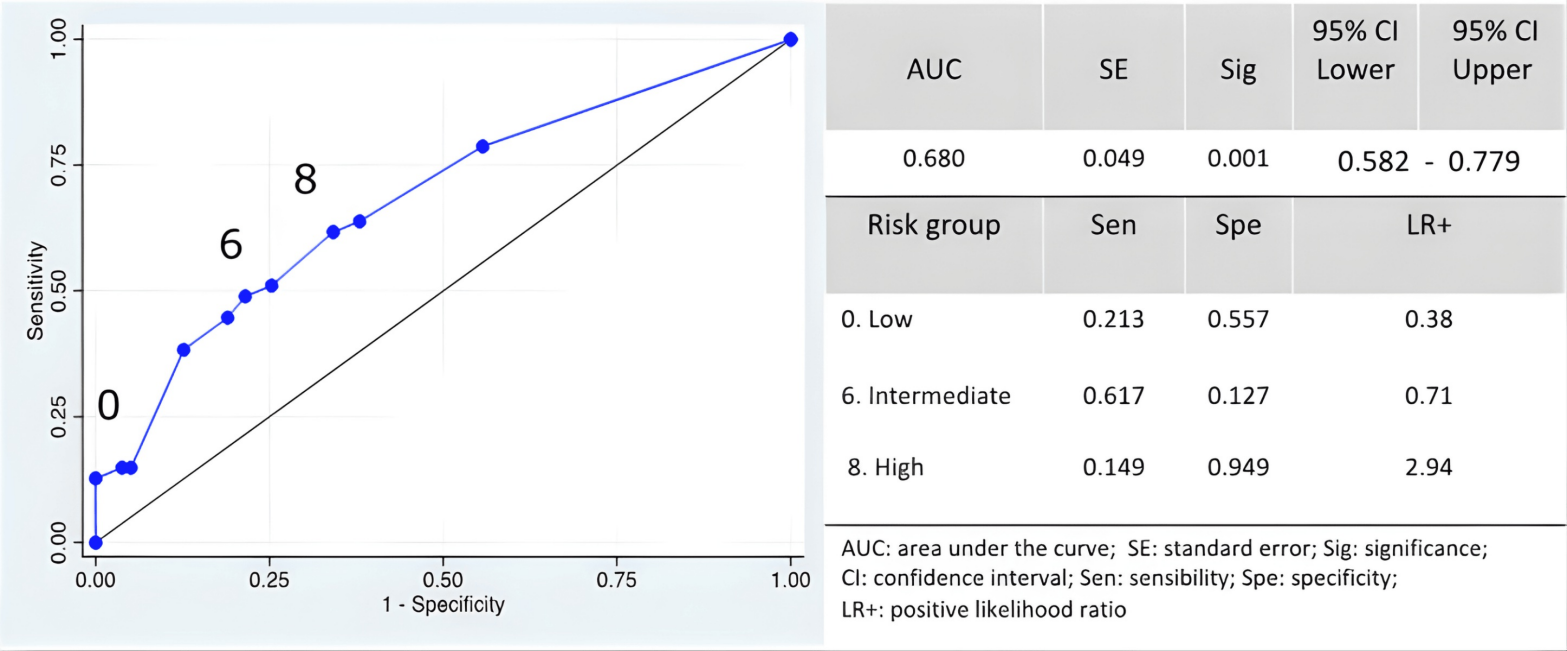
ROC curve of risk factors and the highest likelihood ratio for recurrence.

Four RFs for recurrence were identified with significant clinical relevance: CS IIIB-C, G3 tumors, and LB and HER2+ subtypes. These factors constituted the clinical predictive model, which adequately discriminated the risk of recurrence with an AUC of 0.680 (95% CI: 0.582 to 0.779). The LR+ was 0.38 for LR, 0.71 for IR, and 2.94 for HR, indicating that as LR+ increases, so does the likelihood of recurrence (Figure 2).

Survival analysis

Three non-pCR groups were stratified for final DRFS analysis. Recurrence occurred in 22% (10/45), 36% (19/53), and 64% (18/28) for LR, IR, and HR groups, respectively. The median DRFS for the entire cohort was 38 months (IQR: 23, 50).

**Figure 3:**
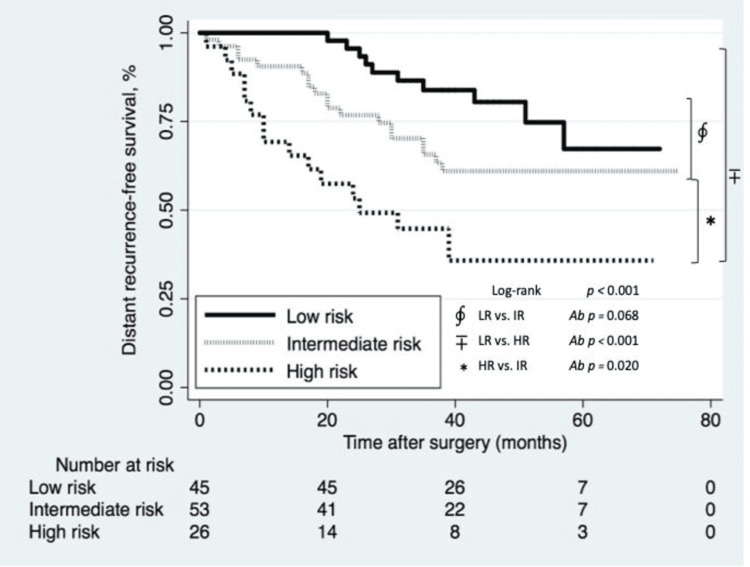
Kaplan-Meier estimates of distant recurrence-free survival according to the scoring system divided into three groups for recurrence risk. Ab: p-value adjusted by Bonferroni correction. Follow-up schedule: Every 4 months (years 1-2), then every 3 months (years 3-5).

Five-year DRFS estimates for LR, IR, and HR groups were 67% (95% CI: 43-83%), 61% (95% CI: 46-73%), and 36% (95% CI: 18-54%), respectively (Figure 3). The LR and IR groups had significantly better five-year DRFS than the HR group (Bonferroni correction, p < 0.001 and p = 0.020, respectively). There was no significant difference between the LR and IR groups (Bonferroni correction, p = 0.068) (Figure 3).

Predictive factors for DRFS

**Figure 4:**
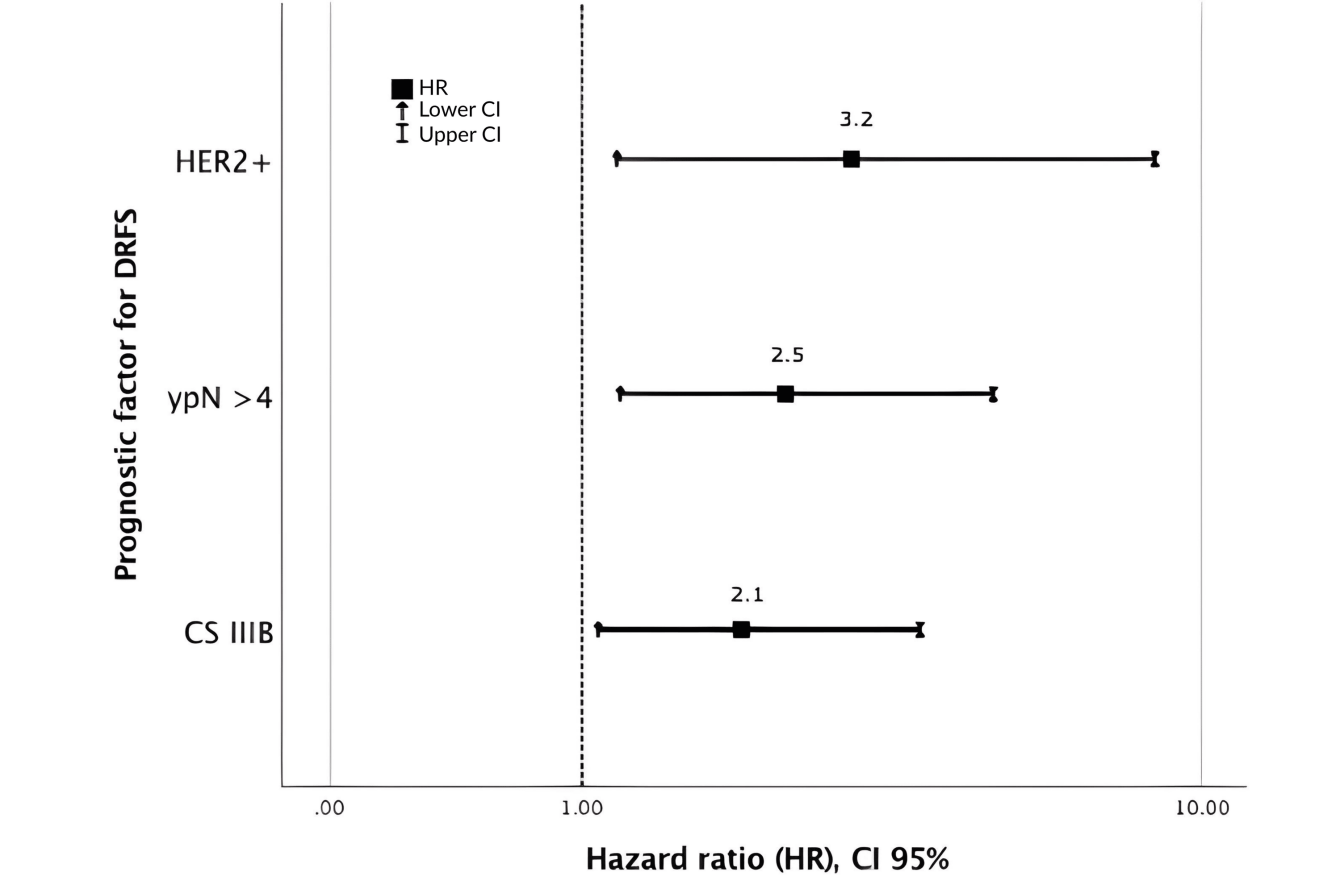
Multivariate analysis of prognostic factors for DRFS. CI: confidence interval, ypN: pathological lymph node; DRFS: distant recurrence-free survival; CS: clinical Stage. Clinical stage was assigned according to the TNM (Tumor, Node, Metastasis) Classification System [15].

Two multivariate analyses were performed, firstly with prognostic factors and secondly with risk groups, both associated with DRFS. As for prognostic factors, multivariate analysis included and confirmed that CS IIIB-C (HR, 2.1; 95% CI: 1.1-4) and HER2+ subtype (HR, 3.2; 95% CI: 1.2-8.6), along with > 4 ypN (HR, 2.5; 95% CI: 1.2-5.2) were significantly associated to DRFS, for this cohort (Figure 4).

As for risk groups, IR (HR, 3.3; 95% CI: 1.2-9) and HR (HR, 5.4; 95% CI: 1.9-15) groups were associated with poor prognosis compared with the LR group (Table 3).

**Table 3:** Cox regression model adjusted by risk groups for distant recurrence-free survival. DRFS: distant recurrence-free survival; β: regression coefficient; SE: standard error; HR: hazard ratio; CI: confidence interval.

Risk group	β	SE	HR	95% CI (Lower)	95% CI (Upper)	P-value
Intermediate risk	1.21	1.71	3.3	1.24	9.1	0.017
High risk	1.69	2.86	5.4	1.93	15	0.001

## Discussion

When a pathological complete response (pCR) occurs following NAC, it is associated with a favorable response in both primary tumor and occult sites with micrometastases, indicating a true sensitivity to the systemic therapy [11]. However, patients with pCR are not exempt from DR after NAC, with rates ranging from 13% to 25% [7, 11]. Two meta-analyses have revealed that pCR alone is insufficient as a surrogate endpoint for DRFS, as the magnitude of response is heterogeneous and varies according to NAC regimen [19, 20]. Prognostic factors are often used to assess the risk of recurrence in patients with pCR or non-pCR after NAC, highlighting the significance of T, N, G [11, 21], biologic markers (ER, HER2) [22, 23], along with molecular subtype [7, 21, 24].

In this study, bivariate analysis identified the clinical significance of several RFs in predicting risk of DR, such as CS IIIB (p = 0.001), G3 tumor (p = 0.001), and LB (p = 0.010), and HER2+ (p = 0.032) breast cancer. When these factors are associated, the likelihood of recurrence increases.

It was also possible to identify those who might have a worse prognosis. For example, if the patient had a CS IIIB and HER2+ breast cancer, they would belong to the HR group (scoring 8) with a poor 5-year DRFS rate of 33% (95% CI: 18-54%). If the patient had a CS IIIB with G3 tumor (scoring 3), they would belong to the IR group with a moderate 5-year DRFS rate of 61% (95% CI: 46-73%). Additionally, patients who had no RFs for recurrence (scoring 0) and belonged to the LR group had a slight increase in their 5-year DRFS rate (67%; 95% CI: 43-83%). Indeed, all patients with residual cancer following NAC are at a high risk of recurrence for metastases [12, 13], since the sum of RFs is associated with a high risk of DR and lower DRFS rates. Gonzalez et al. [11] examined RFs for recurrence in patients with LABC. Their results indicated that premenopausal women with stage IIIB-C breast cancer and fewer than 10 LN examined had a higher risk of developing distant metastases.

Stage is one of the most important prognostic factors in survival [12, 16, 21]. Many Latin American countries with low and middle incomes exhibit poor outcomes in survival and a high mortality rate in women with breast cancer, either due to late diagnosis or lack of screening and treatment programs. When the tumor spreads to distant organs, survival is reduced (24% to 42%) [25, 26]. Breast cancer diagnosed at an advanced stage poses a challenge in oncological management. In Mexico, insured population centers [9] and public health services [3, 26] have provided opportunities to improve survival and quality of life, through the trend toward standardized oncological treatment and the experience of the treating physician.

Fei et al. [27] identified tumor size as the sole predictor of DRFS. Tumors classified as T3 and T4 had an increased risk of distant relapse of 3.6 (95% CI: 1.6-7.8) and 2.8 (95% CI: 0.6-12.6) times, respectively, compared to T1-T2 tumors. In this cohort, five-year DRFS in patients at pathological stage 0-I and with residual tumor at stage IIB-IIIA was 67% (95% CI: 48-81%) and 50% (95% CI: 34-63%), respectively. Thus, extensive residual tumor (T3-T4) carries a high risk of recurrence [11-14].

LN involvement is the most important prognostic factor in survival [12-16, 21], although not as a predictive marker. In this study, DR after NAC occurred in 57% (23/40) of patients who had > 4 ypN, 42% (10/24) with 1-3 ypN, and 23% (14/62) with 0 ypN. Reduced 5-year DRFS rates for groups with > 4 ypN and 1-3 ypN were observed of 35% (95% CI: 17-54%) and 58% (95% CI: 36-75%), respectively. In contrast, patients who had 0 ypN showed a better 5-year DRFS rate of 72% (95% CI: 55-83%; p = 0.001).

The finding that having more than four positive lymph nodes after surgery (>4 ypN) increases the risk of recurrence by 2.5 times is crucial. This reinforces the need for meticulous axillary surgery and detailed pathology. If a patient leaves the operating room with >4 positive lymph nodes, the immediate action is to consider her a candidate for intensive therapy, as NAC failed to eliminate systemic disease in the axilla.

Multivariate analysis confirmed that the number of LN involvement is an independent prognostic factor associated with DRFS. The persistence and number of axillary LN involvement after NAC increase the risk of distant metastases and reduced DRFS rates [12-14, 21]. In fact, ypN staging has greater prognostic weight compared to ypT staging.

Hormone receptor (HRe) status was the first predictive marker introduced in breast cancer and remains an excellent predictor of response to endocrine therapy [21, 23, 28] and is used as a biomarker in molecular classification [7, 21]. There are notable epidemiological, clinical, and molecular differences between ER-positive and ER-negative tumors [7, 24]. Patients with HRe-positive tumors with category RCB 2 or 3 and yAJCC II or III had an intermediate or high risk for recurrence. However, these have a better DRFS rate compared to patients who have the same category in HER2+ or TN breast cancer [12-14].

Luminal tumors, despite having intrinsically lower sensitivity to systemic therapy, recurrence rates appear to be lower and later compared to those who had LB or non-luminal subtypes (TN and HER2+) [7, 10, 29], as observed in our cases, recurrence occurred for LA subtype in 25% and for HER2+ and TN subtypes was 33% and 58%, respectively.

Age and menopause are closely linked. Postmenopausal women with breast cancer exhibit a high incidence of obesity and significantly elevated levels of estrogens (estrone and estradiol) due to the aromatization of androgens into estrogens in adipose tissue, forming an obesity-inflammation-aromatase axis that occurs locally in breast tissue [30]. Approximately more than 70% of postmenopausal patients express bilateral HRe-positive (ER and PgR) with adequate response to hormonal therapy [21, 23, 28].

The breast cancer clinical subtype establishes distinct clinicopathological features in tumor biology [15], aids in guiding targeted therapies [7], and provides predictive and prognostic information [7, 10, 21]. Generally, patients with HER2+ or TN breast cancer tend to experience early recurrences [12, 13]. As for luminal breast cancer, those with the LA subtype tend toward later recurrences compared to those of the LB subtype [29]. The RCB III index identified patients with HER2+ or TN were at high risk of recurrence.

In this study, recurrence varied according to the molecular subtype profile in patients with residual tumor (breast-axilla) after NAC and surgery. LA, LB, HER2+, and TN breast cancer in patients non-pCR experienced DR rates of 27% (10/37), 52% (12/23), 71% (5/7), and 47% (10/21), with poor outcomes for 5-year DRFS of 64% (95% CI: 42-80%), 46% (95% CI: 23-66%), 25% (95% CI: 1-64%) and 47% (95% CI: 23-68%), respectively.

Multivariate analysis confirmed molecular subtype as a prognostic factor associated with DRFS, mainly patients with the HER2+ subtype showed an increased mortality of 3.2 (95% CI: 1.2-8.6) times and a poor 5-year DRFS of 25%, as indicated by other studies [12-14].

HRe-negative tumors are frequently associated with G3 tumors and high Ki-67 indices [11, 23]. These characteristics are often present in HER2+ and TN subtypes [7, 21]. These factors are good predictors of prognosis and response to chemotherapy or even endocrine therapy [7, 10, 23].

In this study, G3 tumors were associated with ER-negative tumors in 55% (n = 24) of cases. As for HRe status, five-year DRFS rate was similar in both patients with ER-positive tumors of 59% (95% CI: 45-70%) and ER-negative tumors of 58% (95% CI: 40-72%; p = 0.430). This finding was confirmed in the multivariate analysis, differences failed to demonstrate statistical significance for ER-negative and G3 tumors (p > 0.05). However, they are clinically relevant factors for the management of systemic therapies.

Not all tumors are sensitive to systemic therapies due to drug-tumor-host resistance mechanisms, refractory tumors [8, 11], or tumor burden in advanced stages, which can limit chemosensitivity. Thus, persistence of residual tumor after NAC increases the probability of DR, as supported by other studies [11-14]

In the clinical setting, it is important to identify patients who achieve less than a pCR to NAC, as they require a close follow-up; a set of pathological indicators and biomarkers should be selected to understand the breast cancer response after NAC and the likelihood of predicting the recurrence risk [10, 12-14].

When breast cancer spreads to distant organs, it becomes an adverse complication; it is often extensive with different clinical scenarios, which makes it difficult to manage with chemotherapy or hormonal treatment alone. In this cohort, it is worth noting that at least two-thirds (69.8%) of patients had residual tumor in the breast and axilla following NAC in patients with LABC. Thus, they were at a high risk of recurrence. Patients who achieved a pCR had a DR of 26% (10 of 38) and 42% (37 of 88) in those with less than a pCR. Therefore, managing targeted therapies remains a challenge to achieve absolute benefit in DRFS, reduce recurrence risk despite molecular subtype profile, improve quality of life, and reduce mortality risks due to comorbidities [6]. Furthermore, the importance of close follow-up from clinical trials is crucial for evaluating the systemic treatment efficacy, identifying recurrence risk after surgery [6, 7, 12], and assessing the potential consequences of residual tumor recurrence.

In this cohort, due to the high prevalence of unfavorable tumor characteristics such as CS IIIB, G3 tumors, non-luminal subtypes (HER2+ and TN), tumor burden (T4), and extensive nodal involvement (> 4 ypN) in patients who did not achieve a pCR, they might increase recurrence and systemic failure rates, as other studies have reported [11-14]

These indicators in the development of the clinical model were associated with poor outcomes due to the high impact of recurrence, even when patients can receive an adequate systemic therapy. Our data are consistent with published studies that have analyzed recurrences and DRFS rates in patients with residual tumor following NAC [11-14].

Limitations

This study has several limitations. First, its retrospective cohort design inherently carries risks of bias. Nevertheless, the cohort was assembled from consecutive patients treated in our service, which reduces selection bias. To address incomplete follow-up, Kaplan-Meier survival curves were generated, with patients lacking full follow-up designated as censored cases.

Second, the study was conducted in a single institution, reflecting only the experience of our hospital. Therefore, external validation in other centers is required before the proposed scale can be generalized.

Third, as a retrospective analysis, the findings reflect treatment patterns that have since evolved. Although modern targeted adjuvant therapies for residual disease are now available, anthracyclines and taxanes remain the backbone of treatment in our setting.

Finally, the limited sample size may affect statistical stability. While the ROC curve demonstrated consistency, three risk strata were necessary to construct multivariate models. This stratification may oversimplify the model; however, it highlights the statistical relevance of the clinical variables most frequently used in our institution.

## Conclusions

Clinical stage IIIB, high histologic grade, and luminal B or HER2-positive subtypes remain robust predictors of distant recurrence in patients with residual breast cancer following neoadjuvant chemotherapy. Clinically, risk stratification into low-, intermediate-, and high-risk groups revealed marked differences in five-year recurrence-free survival, reinforcing the prognostic relevance of these variables.

The clinical implications of our results underscore the critical need for personalized post-neoadjuvant strategies. Patients classified as intermediate- or high-risk should be considered for intensified adjuvant interventions, including extended systemic therapy, incorporation of targeted agents, or enrollment in clinical trials. Furthermore, implementing structured surveillance and multidisciplinary follow-up for these groups may facilitate early detection of relapse and improve outcomes. Integrating these predictors into routine clinical algorithms can optimize therapeutic decision-making and resource allocation, ultimately enhancing long-term survival.
